# Small bowel stenosis following blunt abdominal trauma: a case report

**DOI:** 10.1002/ccr3.1176

**Published:** 2017-09-29

**Authors:** Sheela Prince, Hajer Abdullah Saleh Busharar, Osama Mohemmed AlZoabi

**Affiliations:** ^1^ Department of General surgery Rashid Hospital Dubai UAE

**Keywords:** Blunt abdominal trauma, bowel perforation, bowel stenosis, exploratory laparotomy

## Abstract

Blunt abdominal trauma is a rare case of intestinal obstruction, and only few cases have been reported in the world literature. Stenotic intestinal obstruction following blunt abdominal trauma is a very rare complication**.** This case highlights the need for clinical suspicion serial clinical assessment and radiological evaluation and the need for early surgery in patients presenting with abdominal symptoms *following blunt abdominal trauma*.

## Introduction

Motor vehicle accidents have increasingly became the major cause of blunt abdominal injuries 1, the pattern, and mechanism of which has changed in recent years largely due to seat belt legislation 1. In the absence of shock and peritonism or uncontrolled bleeding, the patients with blunt abdominal injury may be treated conservatively 2, 3 on rare occasions such patients can present later on with features of small bowel obstruction.

## Presentation of Case

A 40‐year‐old male patient was brought to the Accident and Emergency with history of road traffic accident. Patient was examined and managed according to ATLS protocol.

Pulse rate 88 per min, BP 104/84 mm of Hg, SPO2 98%.

Abdomen soft with mild tenderness in both upper quadrants, FAST was positive with collection in the Morrison's pouch and perihepatic area.

**Figure 1:**
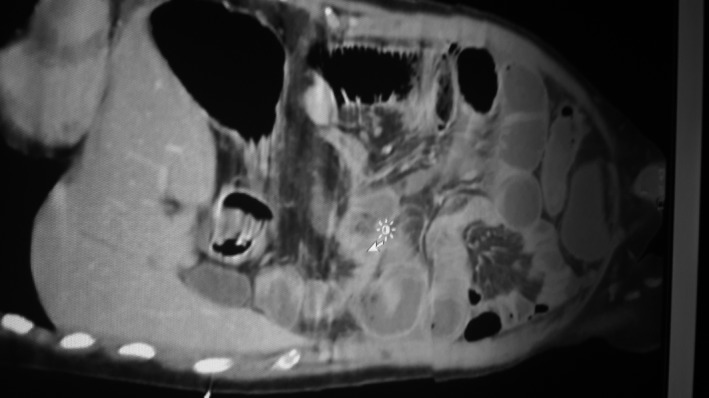
CT Scan abdomen with features of intestinal obstruction.

Polytrauma CT Scan: Brain – there was diffuse cerebral edema.

**Figure 2:**
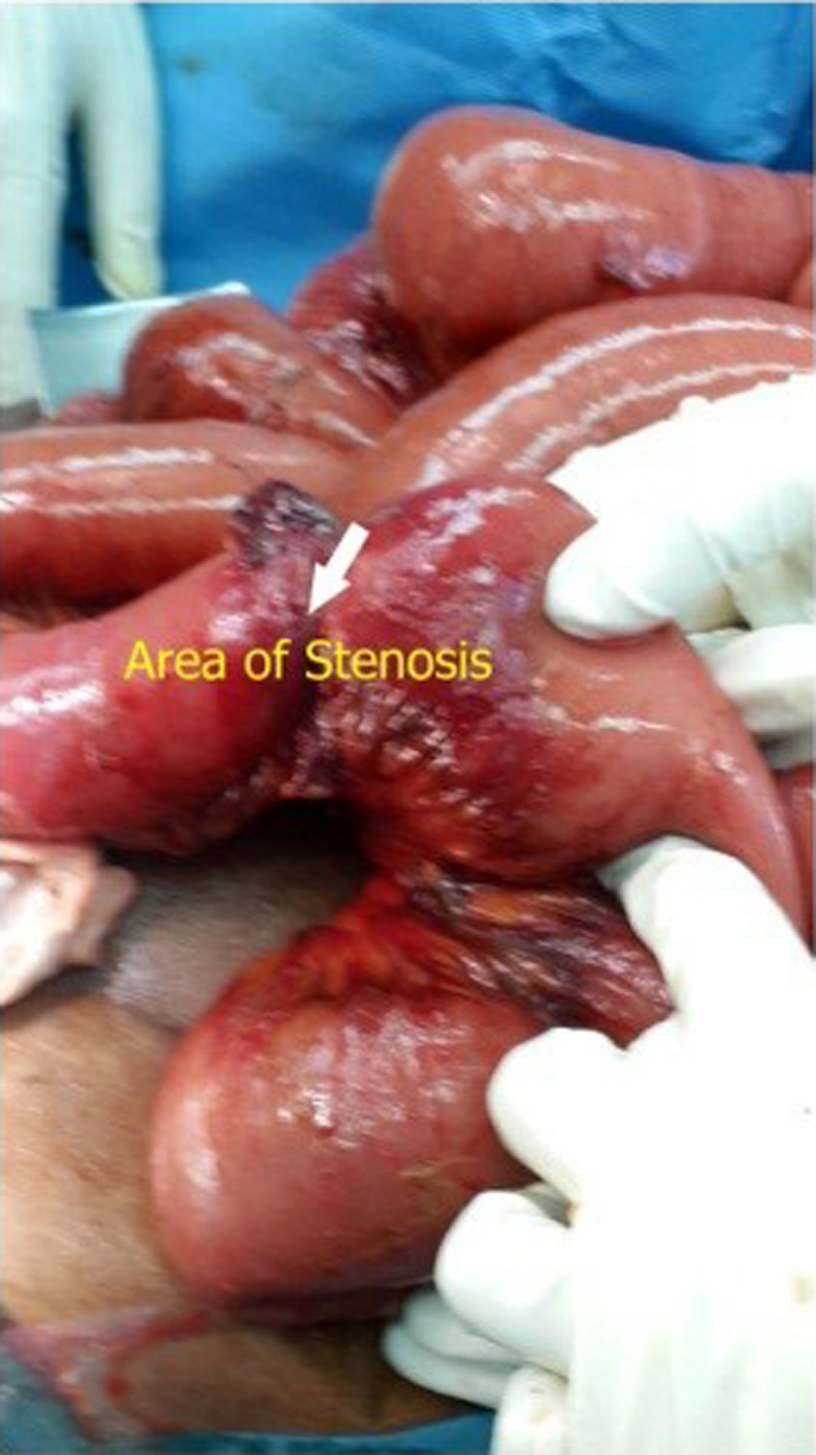
Intraoperative picture of small bowel stenosis.

**Figure 3:**
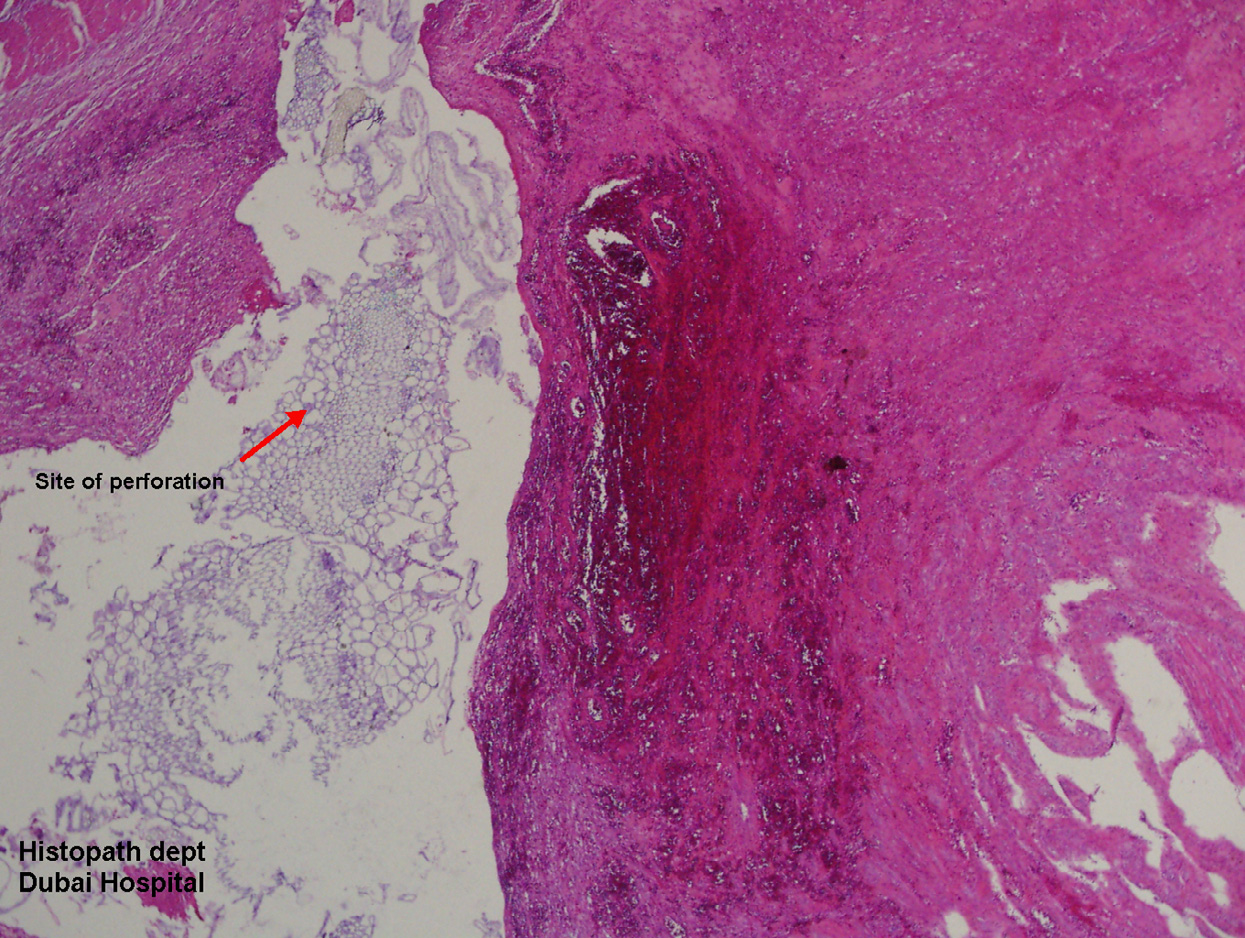
Histopathology of stenosed segment.

Chest, abdomen, and pelvis including thoracic and lumbar spine: There was minimal hemopneumothorax on the right side. Large contusion involves the segments 7, 8, and 4 with minimal perihepatic collection. Contusion is along the superior border of spleen with minimum perisplenic collection. Fracture of transverse process of LI, L2.

X‐Ray left leg: Comminuted fracture of mid shaft of left tibia and fibula.

Blood Investigations: Hb 10.8 gm/dL, HCT 31%, platelet 129x10#3, glucose 114 mg/dL, creatinine .5 mg/dL, urea 17 mg/dL, sodium 139 mmol/L, alkaline phosphatase 310 U/L, ALAT 33 U/bilirubin 1.2 mg/dL, total protein 7.3 g/dL, globulin 3.1 g/dL.

Patient was managed in the surgical ICU. After 2 weeks, he developed increased intra‐abdominal pressure, and laparoscopic drainage of 5 L of bile‐stained fluid was performed and drain was inserted. He underwent nailing of left tibia and discharged in good general condition after a follow‐up CT abdomen after 2 weeks.

On 13.4.2015, patient presented to the Accident and Emergency with 5 days history of abdominal pain, constipation, and nausea. On physical examination, his vital signs were normal. Abdomen distended with hyperactive bowel sounds. Blood investigations including liver function test was normal. Plain X‐ray abdomen was showing distended small bowel loops with multiple air fluid levels. CT scan abdomen was showing dilated small bowel loops involving proximal jejunum and mid ileum with a zone of transition noted in the distal ileum suggestive of intestinal obstruction (Fig. 1). Patient was taken for exploratory laparotomy on the same day. The intraoperative findings were as follows: There was an adhesion band which was attached to the root of mesentery constricting the small bowel 70 cm from the ileocecal junction, and another constricting band of omentum 10 cm above the first producing stenosis of the small bowel. Also there were multiple small omental adhesions of the small bowel to the anterior abdominal wall without producing constriction of the bowel. Release of the adhesions and resection of the stenosed bowel with side‐to‐side anastomosis were performed. Postoperative period was uneventful and discharged after 1 week. Patient was symptom‐free, and all the clinical and biochemical parameters were normal when patient was followed in general surgical clinic after 2 weeks.

Histopathology of resected segment of ileum with stenosis was showing marked inflammation, edema, vascular congestion, and features of perforation.

## Discussion

Abdomen is the third most commonly injured body part following trauma and 85% cases are blunt abdominal trauma 4. Solid organs such as liver and spleen are the most frequently injured organs. In the absence of uncontrolled bleeding or peritonitis, most of the patients are usually managed nonoperatively 3. Small bowel injury has been reported as third most common injury in blunt abdominal trauma 4.

Blunt abdominal trauma is a rare cause of small bowel stenosis producing intestinal obstruction 5, 6. Only few case reports have been described in the world literature 2. The reported time range between blunt abdominal trauma and onset of symptoms ranges from 10 days to 26 years 2, 6. Most of these cases described stenosis or stricture as the cause of Intestinal obstruction 3. One reported late sequelae following blunt abdominal trauma is post‐traumatic intestinal stenosis 6. The etiology of these strictures is usually ischemia secondary to mesenteric tears parallel and close to the involving segment, but, occasionally, direct injury to the bowel wall with subserosal hematoma formation may also subsequent stenosis ileum response to a shearing force 7. In some situations, mesentery is not torn, but direct trauma causes damage to the small bowel results in hemorrhage, mucosal infarction subsequent healing by fibrosis gives rise to cicatricial stenosis and secondary small bowel obstruction 7. The pathology was either a stricture or old perforation or adhesions causing intestinal obstruction 8.

Common clinical symptoms of small bowel obstruction nausea, abdominal pain and vomiting or vague abdominal symptoms to frank obstruction 1, 6. Diagnosis of small bowel stenosis following blunt abdominal trauma is achieved by proper clinical examination, plain X‐ray abdomen, and contrast‐enhanced CT scan of abdomen confirmatory. Treatment is immediate surgical intervention after confirmation of diagnosis. In our case, blunt abdominal trauma caused small bowel stenosis and delayed Intestinal obstruction after 7 weeks. There was evidence of perforation in the histological specimen examined from resected stenosed segment of small bowel. Laparotomy and resection anastomosis and adhesiolysis were curative.

## Conclusion

Post‐traumatic small bowel stenosis is a clinical entity that needs to be watched for in all patients presenting with abdominal symptoms following blunt abdominal trauma managed conservatively.

## Consent

Written informed consent obtained from the patient for publication of this case report and accompanying images.

## Authorship

SP: was primary surgeon in this surgery. HASB: assisted the surgery. OMAZ: assisted in laparoscopic drainage.

## Conflict of Interest

None declared.
